# A Simple Nomogram to Predict Contrast-Induced Acute Kidney Injury in Patients with Congestive Heart Failure Undergoing Coronary Angiography

**DOI:** 10.1155/2021/9614953

**Published:** 2021-03-23

**Authors:** Li Lei, Yibo He, Zhaodong Guo, Bowen Liu, Jin Liu, Zhiqiang Nie, Guanzhong Chen, Liwei Liu, Mengfei Lin, Wenhe Yan, Shiqun Chen, Chen Jiyan, Yong Liu

**Affiliations:** ^1^The Second School of Clinical Medicine, Southern Medical University, Guangzhou, Guangdong, China; ^2^Department of Cardiology, Provincial Key Laboratory of Coronary Heart Disease, Guangdong Cardiovascular Institute, Guangdong Provincial People's Hospital Affiliated with South China University of Technology, Guangdong Academy of Medical Sciences, Guangzhou, Guangdong, China; ^3^Guangdong Provincial People's Hospital, School of Medicine, South China University of Technology, Guangzhou, Guangdong, China; ^4^Department of Cardiology, Maoming People's Hospital, Maoming, Guangdong, China

## Abstract

**Background:**

Patients with congestive heart failure (CHF) are vulnerable to contrast-induced kidney injury (CI-AKI), but few prediction models are currently available. Therefore, we aimed to establish a simple nomogram for CI-AKI risk assessment for patients with CHF undergoing coronary angiography.

**Methods:**

A total of 1876 consecutive patients with CHF (defined as New York Heart Association functional class II-IV or Killip class II-IV) were enrolled and randomly (2:1) assigned to a development cohort and a validation cohort. The endpoint was CI-AKI defined as serum creatinine elevation of ≥0.3 mg/dL or 50% from baseline within the first 48–72 hours following the procedure. Predictors for the simple nomogram were selected by multivariable logistic regression with a stepwise approach. The discriminative power was assessed using the area under the receiver operating characteristic (ROC) curve and was compared with the classic Mehran score in the validation cohort. Calibration was assessed using the Hosmer–Lemeshow test and 1000 bootstrap samples.

**Results:**

The incidence of CI-AKI was 9.06% (170) in the total sample, 8.64% (*n* = 109) in the development cohort, and 9.92% (*n* = 61) in the validation cohort (*P*=0.367). The simple nomogram including four predictors (age, intra-aortic balloon pump, acute myocardial infarction, and chronic kidney disease) demonstrated a similar predictive power as the Mehran score (area under the curve: 0.80 vs. 0.75, *P*=0.061), as well as a well-fitted calibration curve.

**Conclusions:**

The present simple nomogram including four predictors is a simple and reliable tool to identify CHF patients at risk of CI-AKI, whereas further external validations are needed.

## 1. Introduction

Contrast-induced kidney injury (CI-AKI) is a common complication following coronary angiography (CAG) or percutaneous coronary intervention (PCI), and the occurrence of CI-AKI has been demonstrated to be related to poor outcomes [1–3]. Published guidelines recommend hydration therapy during the perioperative period of coronary catherization as one of the important strategies to prevent CI-AKI [4, 5]. However, for patients with congestive heart failure (CHF), the extra fluid load might lead to further adverse events of acute heart failure [6, 7], even though hydration volume is halved in such patients. Therefore, precise stratification and identification of patients at high risk of CI-AKI are required to perform preventive hydration accurately to reduce unnecessary overload complications. Previous studies have reported various prediction models for CI-AKI, but few of them were developed for CHF patients specifically, even though CHF patients are vulnerable to CI-AKI [8]. In addition, the classic Mehran score, which includes 8 variables, might be too complicated for clinical application [9]. Therefore, in this study, we aimed to establish a simple nomogram for CI-AKI risk assessment among patients with CHF undergoing CAG/PCI.

## 2. Materials and Methods

### 2.1. Patients

The current study population was based on a prospective observation cohort (PREdictive Value of COntrast voluMe to creatinINe Clearance Ratio, PRECOMIN, NCT01400295) which enrolled consecutive patients undergoing CAG/PCI in Guangdong Provincial People's Hospital between January 2010 and October 2012. In this study, patients with CHF (defined as New York Heart Association (NYHA) functional class II-IV or Killip class II-IV) undergoing CAG/PCI were enrolled. [10] The exclusion criteria were pregnancy, lactation, contrast exposure within the 7 days before or 3 days after the procedure, cardiovascular surgery, no use of low-osmolarity contrast agents, undergoing hemodialysis, missing preoperative or postoperative creatinine, malignancy, and no use of isotonic saline for hydration). The Ethics Committee of the Guangdong Provincial People's Hospital approved this study. All the patients involved provided written informed consent and were randomly assigned to a development cohort and a validation cohort in a 2:1 ratio.

### 2.2. Coronary Angiography and Laboratory Examination

The procedures were conducted by interventional cardiologists following published guidelines, institutional policy, and clinical routine. [11] Patients undergoing a non-emergency procedure received hydration therapy (0.5–1 mL/kg/h for at least 2–12 hours before and 6–24 hours after the procedure). Patients undergoing emergency procedures received unspecified hydration therapy before the procedure. Serum creatinine (SCr) was measured for all patients at admission and at 1, 2, and 3 days after the procedure using the Jaffe method.

### 2.3. Endpoint and Definitions

The endpoint of this study was CI-AKI defined as a SCr elevation of ≥0.3 mg/dL or 50% from baseline within the first 48 to 72 hours following contrast exposure. [12] Baseline characteristics, angiographic data, and medications were prospectively defined and have been reported in a previous study [13]. The definitions of hypotension, diabetes, anemia, chronic kidney disease (CKD), and intra-aortic balloon pump (IABP) were the same as those used for the Mehran score [9]. Acute myocardial infarction (AMI) was defined according to the third universal definition of myocardial infarction [14]. Hypoalbuminemia was defined as serum albumin <35 g/L [15]. Age, weight, heart rate (HR), and contrast volume were defined as continuous variables. Angiotensin-converting enzyme inhibitors (ACEIs)/angiotensin-receptor blockers (ARBs), diuretics, and beta blockers were defined as the prescription of these medicines during the perioperative period. To calculate the estimated glomerular filtration rate (eGFR), the Modification of Diet in Renal Disease (MDRD) equation was used: eGFR (mL/min/1.73 m^2^) = 186 *∗* (SCr ^  − 1.154) *∗* (Age ^  − 0.203) *∗* 0.742 (if female). The unit of SCr in this formula is mg/dl. CKD was defined as eGFR <60 mL/min/1.73 m^2^. Patients were further divided into 5 CKD stages according to the guidelines (CKD G1: eGFR ≥ 90 mL/min/1.73 m^2^; CKD G2: eGFR: 60–90 mL/min/1.73 m^2^; CKD G3: eGFR: 30–60 mL/min/1.73 m^2^; CKD G4: eGFR: 15–30 mL/min/1.73 m^2^; and CKD G5: eGFR: <15 mL/min/1.73 m^2^). [16] And they were also divided into 3 categories according to their heart function based on NYHA or Killip class. All eligible patients enrolled were followed up at 1 month, 6 months, and every 1 year after enrollment until April 2019.

### 2.4. Statistical Analysis

All the patients involved were randomly assigned to a development cohort and a validation cohort in a 2:1 ratio. Continuous variables were compared with an unpaired, 2-tailed *t*-test and are expressed as the mean ± SD or were compared through the Wilcoxon rank-sum test and are expressed as the median ± interquartile. Categorical variables were compared using the *χ*^2^ test or Fisher's exact test and are expressed as percentages. Kaplan–Meier curve and multivariable Cox proportional hazard regression adjusted for known risk factors in the context of long-term prognosis were performed to explore the association between CI-AKI and long-term mortality. [17, 18].

To build the nomogram, candidate variables that were imbalanced between groups in the development cohort or that are clinically important, such as risk factors included in the traditional Mehran score, were included in the univariable logistic analysis. Variables with >15% missing values were not considered candidates, i.e., low-density lipoprotein-C and HbA1c. Significant variables from the univariable logistic analysis were then included in the multivariable logistic analysis. A backward stepwise approach was performed to screen the variables by successively removal of nonsignificant (*P* < 0.1) covariates until all the remaining variables were statistically significant. Then, we manually investigated the contribution of the remaining variables to determine the final predictors. Collinearity between variables was also evaluated. A nomogram was then formulated based on the results and by using the rms package of R. To form the nomogram, each regression coefficient in the multivariate logistic regression was proportionally converted into a 0- to 100-point scale. The variable with the highest *β* coefficient (absolute value) was assigned 100 points. The points are added across each variable to calculate the total points, which are finally converted to predicted probabilities. The performance of the nomogram was assessed using the area under the receiver operating characteristic (ROC) curve and concordance C-statistic for discriminative ability. For validation, a bootstrap method (1000 times) was performed in both the development and validation cohorts to evaluate the stability of the C-statistic. Calibration was assessed using the Hosmer–Lemeshow test and the 1000 bootstrap samples to decrease the overfit bias [19, 20]. Area under curve (AUC) comparison between the nomogram and the Mehran score was performed using DeLong's test in the validation cohort. Missing data were not imputed. In all analyses, *P* < 0.05 was considered statistically significant. All analyses were conducted with *R* software (version 3.6.2; *R* Foundation for Statistical Computing, Vienna, Austria) and SPSS (version 26.0).

## 3. Results

### 3.1. Baseline Characteristics

The details and missing data of the included patients are listed in Supplementary Table 1. Among the included 1876 CHF patients, 1261 and 615 patients were divided into the development and validation cohorts, respectively. In the total cohort, approximately one-fourth were female (25.11%). The mean age was 64.77 ± 10.70 years, and the mean SCr was 96.86 ± 50.73 *μ*mol/L. No significant differences were identified between the development and validation cohorts, except for the diastolic blood pressure, contrast volume, and the proportion of percutaneous coronary intervention (PCI) during the procedure.

The incidence of CI-AKI was 9.06% (*n* = 170) in the total sample, 8.64% (109 patients) in the development cohort, and 9.92% (61 patients) in the validation cohort (*P*=0.367). The distribution of the SCr value within the time frame (24–72 hours after the procedure) is shown in Supplementary Figure 1. For the development cohort, patients complicated with CI-AKI following CAG tended to be older and had lower left ventricular ejection fraction (LVEF), haemoglobin, and eGFR than those without CI-AKI. Patients with CI-AKI were also more likely to have hypotension, diabetes, CKD, AMI, and intra-aortic balloon pump (IABP), and they were less likely to be prescribed ACEI/ARB and beta blockers. No significant difference between groups was identified in contrast volume (Table 1).

### 3.2. Development and Validation of the CI-AKI-Predicting Nomogram

The results of univariate logistic analysis are detailed in Table 2. Through multivariate logistic analysis and a backward stepwise approach, age (OR: 1.04 95%, CI: 1.02–1.06), IABP (OR: 3.97 95%, CI: 2.14–7.37), AMI (OR: 3.27 95%, CI: 2.09–5.10), and CKD (OR: 2.83 95%, CI: 1.80–4.44) were selected as predictors of CI-AKI (Table 3). The Hosmer–Lemeshow statistic of multivariable analysis suggested a good fit (*χ*^2^ = 10.78, *P*=0.214).

**Figure 1:**
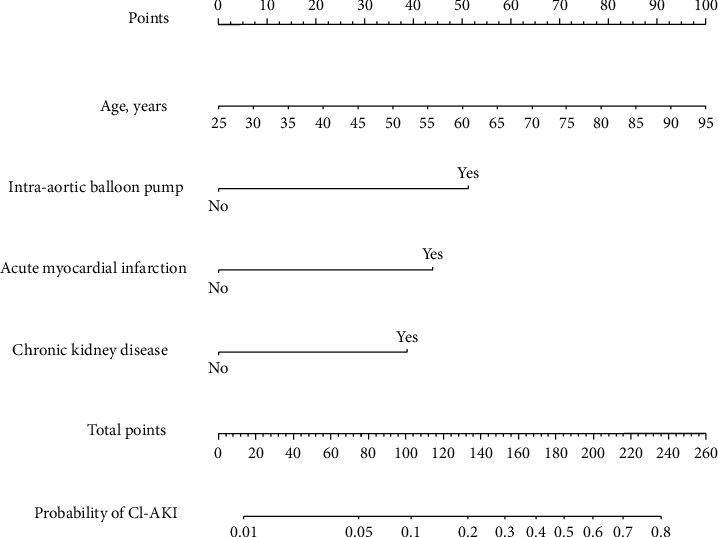
Nomogram to estimate the risk of CI-AKI. To use the nomogram, find the position of each variable on the relative axis, draw a line to the points axis for the number of points, add the points derived from all the variables together, and refer to the total points axis to determine CI-AKI probability. For example, a 60-year-old man, with acute myocardial infarction and chronic kidney disease, underwent intra-aortic balloon pump periprocedure. The expected CI-AKI probability with the nomogram is 60 years old = 50 points; AMI = 44 points; IABP = 51 points; and CKD = 39 points. Total 184 points; predicted CI-AKI probability ≈49.7%.

**Figure 2:**
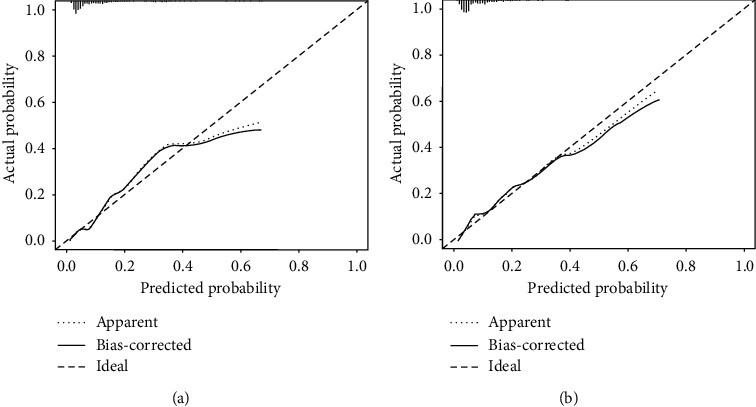
(a) Validity of the predictive value of the nomogram for the estimation of the risk of CI-AKI in the development cohort. (b) Validity of the predictive value of the nomogram for the estimation of the risk of CI-AKI in the validation cohort.

A simple nomogram based on the selected predictors was formed (Figure 1). The nomogram was internally validated with the bootstrap validation method (1000 times). In the development cohort, the nomogram demonstrated good discriminative power for estimating the risk of CI-AKI, with an unadjusted C-statistic of 0.79 (95% CI, 0.75–0.84) and a bootstrap-corrected C statistic of 0.79. In addition, calibration plots graphically showed good agreement on the presence of CI-AKI between the risk estimation and the observed frequency (Figure 2(a)).

**Figure 3:**
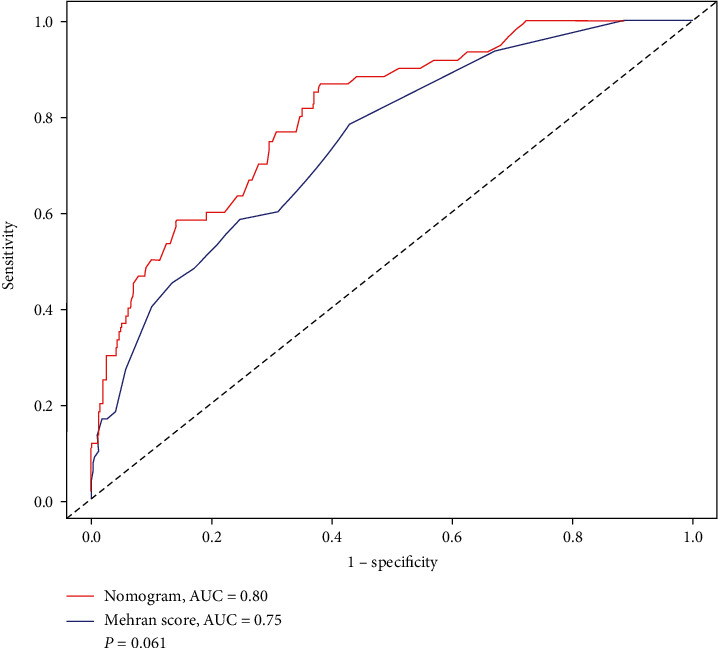
The receiver operator characteristic curves of the nomogram and Mehran score in the validation cohort.

In the validation cohort, the simple nomogram demonstrated a C-statistic of 0.80 (95% CI 0.75–0.86), which was similar to that of the Mehran score (AUC: 0.75, 95% CI 0.68–0.81) among patients with CHF (*P*=0.061, Figure 3) and a bootstrap-corrected C statistic of 0.80. Moreover, there was also a good calibration curve for the risk estimation (Figure 2(b)).

The discriminative ability and calibration were also tested among patients with different stages of CHF and stages of CKD, respectively. No significant difference in discriminative ability was identified, except for the comparison between “Heart function II” and “Heart function IV.” Hosmer–Lemeshow statistic suggested a good fit in all stages of CHF and stages of CKD (Supplementary Table 2).

### 3.3. Risk of CI-AKI Based on the Nomogram Scores

Based on the predicted incidence of CI-AKI in relation to different total nomogram scores, we further divided the patients into 5 score categories: scores < 50 (risk = 1.96%), 50 ≤ scores < 100 (risk = 4.71%), 100 ≤ scores < 150 (risk = 13.90%), 150 ≤ scores < 200 (risk = 39.09%), and scores ≥ 200 (risk = 65.78%). The rates of CI-AKI in the validation cohort were close to those in the development cohort inside each of the 5 score categories (Supplementary Figure 2).

The optimal cut-off value of the total nomogram scores to identify patients at risk was determined to be 100. The sensitivity and specificity used to differentiate the presence from the absence of CI-AKI were 69.7% and 75.6% in the development cohort and 70.0% and 74.5% in the validation cohort, respectively. The total nomogram scores of 100 to identify patients at risk also demonstrated a good discriminative power in both the development cohort (AUC: 0.73, 95% CI: 0.68–0.78, and *P* < 0.001) and the validation cohort (AUC: 0.72, 95% CI: 0.65–0.79, and *P* < 0.001).

## 4. Discussion

The present study might be the first to develop a simple nomogram for the prediction of CI-AKI among CHF patients undergoing CAG/PCI. Through multivariable logistic regression analysis and a stepwise approach, a nomogram based on four factors (age, IABP use, AMI, and CKD) was constructed. In particular, the simple nomogram demonstrated a similar discriminative power as the classic Mehran score in the validation cohort, as well as good stability and calibration.

To define CHF patients in this study, two classification methods (NYHA and Killip) were used interchangeably. Previous studies had already taken this method to identify CHF patients [21, 22]. In our clinical routine, we evaluate the heart function of AMI and non-AMI patients with Killip and NYHA system, respectively. Using these two systems interchangeably may help identifying patients with impaired heart function more concisely.

In this study of the CHF population, the overall incidence of CI-AKI was 9.06%. The previously reported incidence of CI-AKI has not been consistent among different studies. Among the patients with a low-risk of ordinary coronary artery disease, the CI-AKI incidence was as low as 2-3%, while in the high-risk population, the CI-AKI incidence could be as high as 19% or 50% [2, 3]. Since heart failure patients might be complicated with haemodynamic disturbances, which might further cause kidney dysfunction [23, 24], the relatively high incidence of CI-AKI among our CHF cohort was mostly rational. Moreover, we noticed that some patients were missing SCr data on day 3, which may lead to the underestimation of the incidence of CI-AKI. However, many previous studies had limited the diagnosis time frame of CI-AKI to 48 hours, or even 24 hours [9, 25]. In the study conducted by Kim et al., CI-AKI was defined as an increase in SCr of ≥50% or 0.3 mg/dL, or a decrease in eGFR of ≥25% within 24 hours after PCI [25].

The present simple nomogram was constructed based on age, AMI, IABP, and CKD, which are common risk factors associated with CI-AKI, as reported by numerous previous studies [12, 26, 27]. Thomas T. Tsai et al. conducted a study including 985,737 consecutive patients who underwent PCI at 1,253 sites in America and found that ST-segment elevation myocardial infarction (STEMI) (OR: 2.60; 95% CI: 2.53–2.67) as well as non-ST-segment elevation myocardial infarction (NSTEMI) (OR: 1.81; 95% CI: 1.61–2.04) were independently associated with CI-AKI. Additionally, each 10-year increase in age (OR: 1.15; 95% CI: 1.14–1.16), IABP before the procedure (OR: 2.13; 95% CI: 1.92–2.35), and CKD stage 3–5 were all risk factors for CI-AKI [2]. Pierre Aubry et al. performed a retrospective cross-sectional population-based study involving 1,047,329 cases of contrast exposure in Europe to quantify the effect of risk factors for CI-AKI. By multivariate analysis, age >80 years (OR: 2.7; 95% CI: 2.6–2.8) and CKD (OR: 2.3; 95% CI: 2.2–2.3) were identified as independent risk factors for CI-AKI [28]. For patients in Asia, Pei-Chun Fan et al. developed and validated a risk prediction model (ADVANCIS score) for incident CI-AKI based on 82,186 patients admitted for acute coronary syndrome (ACS) receiving PCI. Through multivariable logistic regression analysis, age (OR: 1.02; 95% CI: 1.01–1.02), CKD (OR: 11.38; 95% CI: 9.86–13.13), and IABP use (OR: 1.80; 95% CI: 1.62–2.00) were strongly associated with the incidence of CI-AKI. [29].

The nomogram developed in this study is a simple but efficient prediction model that includes only four predictors. We noticed that some important risk factors reported by previous results were not included in the current simple nomogram, such as contrast volume and diabetes [30]. The nonsignificant difference between the CI-AKI and non-CI-AKI subjects in contrast volume may be one of the reasons. In addition, some recent studies have found that the dose of contrast agent is not necessarily related to the incidence of CI-AKI [31, 32]. Also, with the development of coronary interventional treatment techniques, the volume of contrast agents is gradually reduced [9]. Moreover, in the current study, all patients included were restricted to use low-osmolarity contrast agents during the procedure, and the risk of kidney impairment was reduced by the usage of low-osmolarity and isotonic contrast agents [33]. The risk of CI-AKI might be due to haemodynamic instability rather than direct injury by contrast agents. Regarding diabetes mellitus, a long course of hyperglycaemia would result in renal microcirculation disorder and renal dysfunction, which might affect the incidence of CI-AKI [34, 35]. However, there is no evidence suggesting that short-term hyperglycaemia associated with diabetes mellitus without renal dysfunction would increase the risk of CI-AKI. Therefore, the novel model we developed directly included CKD as one of the predictors rather than diabetes mellitus. Moreover, adding contrast volume and diabetes to the nomogram did not significantly increase the C-statistic (>10%).

The Mehran score is one of the most classic CI-AKI risk estimating system, which was developed based on 8,357 patients undergoing PCI [9]. Comparing with the Mehran score, the current nomogram has several strengths. First, our previous study showed that the CI-AKI definition of this study has higher population attributable risk (PAR) than that of the CI-AKI definition of the Mehran score, which means the current CI-AKI definition has better prognostic value [36]. Second, the current nomogram with only 4 variables was established for CHF patients specifically. Less variables lead to easier clinical implication. However, the nomogram also has some shortness. First, the nomogram was developed based on a relatively small cohort. Second, the nomogram lacks external validation.

We also noticed that several researches have provided nomograms to predict acute kidney injury (AKI) lately. However, these nomograms were developed for different patients specifically [37–39]. By analysing the Medical Information Mart for Intensive Care- (MIMIC-) III v. 1.4 database, Deng et al. established a nomogram to predict the risk of septic AKI within the first 24 h after admission of intensive care unit. Among the included 2,917 sepsis patients without CKD, the nomogram with 7 variables demonstrated well-fitted calibration curves and good C-indexes in both the training and validation cohorts [37]. In another study conducted by Xu et al., they developed a preprocedural nomogram with 5 variables to predict the risk of AKI among patients undergoing nephrectomy [38]. Comparing with these nomograms, our nomogram was developed specifically for the CHF population undergoing CAG/PCI. Moreover, with fewer and readily accessed variables, our nomogram was easier for clinical implication in the department of cardiology.

Overall, in this study, we established a novel CI-AKI risk prediction nomogram specified for the CHF population. In this simple nomogram, patients with more than 100 total points are identified as at-risk patients, accounting for nearly 30% of the entire CHF cohort. The threshold of 100 points (approximately 10% risk) was also similar to the risk threshold of previous models [9, 29].

### 4.1. Limitations

Our study had some limitations. First, this study was an analysis of single-centre data and the nomogram we established is not a general one for CI-AKI risk evaluation in any radiological study, but specifically for coronary study/intervention. However, no relevant study had so far elucidated the specified risk factors of CI-AKI in CHF patients. Our study provided a new tool for CI-AKI risk assessment among CHF patients, prompting more attention and further studies on risk assessment of patients with comorbidities undergoing coronary catheterization. Second, the present nomogram was not externally validated, although there was a calibration with 1000 bootstrap samples to decrease the overfit bias. Third, our nomogram did not demonstrate better discriminative power than the Mehran score. However, our model has less variables, which makes it easier for clinical implement. Fourth, some other potential risk factors, especially procedural context (i.e., emergency vs. elective procedure), were not included due to the insufficient data. Finally, some patients were discharged within 72 hours after the CAG/PCI, so creatinine levels were not measured on day 3 in these patients, which might lead to the underestimation of the incidence of CI-AKI.

## 5. Conclusions

The presented nomogram with four predictors (age, IABP, AMI, and CKD) is a simple and reliable tool for CI-AKI risk stratification among CHF patients, which enables physicians to identify patients at risk and implement precise prevention strategies in time. However, further external validations are needed before clinical generalization.

## Figures and Tables

**Table 1:** Baseline characteristics in the development cohort.

Variables	Missing value, *n* (%)	CI-AKI (*n* = 109)	Non-CI-AKI (*n* = 1152)	*P* value
*Age, years*	0 (0)	69.72 ± 10.75	64.24 ± 10.36	<0.001
** **Age ≥ 65 years, *n* (%)	0 (0)	80 (73.39)	584 (50.69)	<0.001
** **Age ≥ 75 years, *n* (%)	0 (0)	41 (37.61)	203 (17.62)	<0.001

Female sex, *n* (%)	0 (0)	33 (30.28)	281 (24.39)	0.175
Weight, kg	8 (0.63)	62.46 ± 9.84	64.70 ± 10.93	0.027
SBP, mmHg	3 (0.24)	128.83 ± 28.19	130.77 ± 20.39	0.487
DBP, mmHg	4 (0.32)	73.64 ± 12.70	76.54 ± 11.98	0.024
HR, bpm	3 (0.24)	80.04 ± 16.94	74.86 ± 13.72	0.003

*Medical history*				
** **Hypotension, *n* (%)	4 (0.32)	11 (10.28)	25 (2.17)	<0.001
** **CKD, *n* (%)	0 (0)	57 (52.29)	236 (20.49)	<0.001
** **CKD stages	0 (0)			<0.001
** **CKD G1, *n* (%)		14 (12.84)	358 (31.08)	
** **CKD G2, *n* (%)		38 (34.86)	558 (48.44)	
** **CKD G3, *n* (%)		41 (37.61)	221 (19.18)	
** **CKD G4, *n* (%)		16 (14.68)	13 (1.13)	
** **CKD G5, *n* (%)		0 (0)	2 (0.17)	
** **LVEF, %	137 (10.86)	50.36 ± 12.54	57.50 ± 13.15	<0.001
** **LVEF < 40%, *n* (%)	137 (10.86)	20 (19.80)	123 (12.02)	0.025

*Heart function (NYHA class)*	0 (0)			<0.001
** **II, *n* (%)		28 (25.69)	742 (64.41)	
** **III, *n* (%)		14 (12.84)	98 (8.51)	
** **IV, *n* (%)		3 (2.75)	15 (1.30)	

*Heart function (Killip class)*	0 (0)			<0.001
** **II, *n* (%)		42 (38.53)	250 (21.70)	
** **III, *n* (%)		10 (9.17)	34 (2.95)	
** **IV, *n* (%)		12 (11.01)	13 (1.13)	

Hypertension, *n* (%)	1 (0.08)	85 (77.98)	703 (61.08)	<0.001
Hyperlipidemia, *n* (%)	0 (0)	16 (14.68)	166 (14.41)	0.939
Hypoalbuminemia, *n* (%)	108 (8.56)	60 (68.18)	479 (44.98)	<0.001
Anemia, *n* (%)	21 (1.67)	54 (50.47)	378 (33.36)	<0.001
AMI, *n* (%)	4 (0.32)	64 (58.72)	297 (25.87)	<0.001
Diabetes, *n* (%)	0 (0)	38 (34.86)	293 (25.43)	0.032
CAD, *n* (%)	6 (0.48)	104 (95.41)	1068 (93.19)	0.373

*Laboratory examination*				
** **LDL-C, mmol/L	224 (17.76)	2.94 ± 1.10	2.67 ± 0.95	0.045
** **HDL-C, mmol/L	224 (17.76)	0.96 ± 0.30	1.05 ± 2.20	0.267
** **SCr, *μ*mol/L	0 (0)	120.76 ± 55.33	93.12 ± 44.14	<0.001
** **eGFR, ml/min/1.73 mm^2^	0 (0)	63.16 ± 34.49	78.88 ± 23.74	<0.001
** **Hemoglobin, g/L	73 (5.79)	122.19 ± 21.61	133.06 ± 16.42	<0.001
** **HbA1c, %	270 (21.41)	6.97 ± 1.59	6.59 ± 1.36	0.040

*Medications*				
** **ACEI/ARB, *n* (%)	0 (0)	87 (79.82)	1004 (87.15)	0.032
** **Beta blocker, *n* (%)	1 (0.08)	68 (62.39)	992 (86.19)	<0.001
** **Statin, *n* (%)	0 (0)	102 (93.58)	1113 (96.61)	0.109
** **Diuretics, *n* (%)	1 (0.08)	52 (47.71)	236 (20.50)	<0.001

*Procedure*				
** **PCI, *n* (%)	61 (4.84)	71 (78.02)	782 (70.51)	0.129
** **Hydration volume, mL	32 (1.71)	1130.31 ± 674.82	788.37 ± 433.57	<0.001
** **Contrast volume, mL	0 (0)	139.72 ± 72.85	133.88 ± 67.78	0.422
** **Contrast volume ≥ 100 mL, *n* (%)	0 (0)	81 (74.31)	869 (74.43)	0.795
** **Contrast volume ≥ 200 mL, *n* (%)	0 (0)	21 (19.27)	224 (19.44)	0.964
** **Mehran score	23 (1.82)	9.75 ± 6.19	4.72 ± 4.18	<0.001
** **Peri-procedure IABP, *n* (%)	0 (0)	26 (23.85)	35 (3.04)	<0.001

CI-AKI: contrast-induced acute kidney injury; SBP: systolic blood pressure; DBP: diastolic blood pressure; HR: heart rate; LVEF: left ventricular ejection fraction; CKD: chronic kidney disease; NYHA: New York Heart Association; AMI: acute myocardial infarction; CAD: coronary artery disease; LDL-C: low-density lipoprotein-C; HDL-C: high-density lipoprotein-C; SCr: serum creatinine; eGFR: estimate glomerular filtration rate; ACEI: angiotensin-converting enzymes inhibitors; ARB: angiotensin-receptor blockers; PCI: percutaneous coronary intervention; and IABP: intra-aortic balloon pump.

**Table 2:** Univariable logistic regression analysis of CI-AKI.

Variable	OR (95% CI)	*P* value
Age, years	1.06 (1.04–1.08)	<0.001
Age ≥ 65 years vs. age < 65 years	2.68 (1.73–4.17)	<0.001
Age ≥ 75 years vs. age < 75 years	2.82 (1.86–4.27)	<0.001
Weight, kg	0.98 (0.96–1.00)	0.040
HR, bpm	1.02 (1.01–1.04)	<0.001
DBP, mmHg	0.98 (0.96–1.00)	0.017
CKD vs. no CKD	4.25 (2.85–6.36)	<0.001
SCr, *μ*mol/L	1.01 (1.00–1.01)	<0.001
eGFR, ml/min/1.73 mm^2^	0.97 (0.96–0.98)	<0.001
LVEF<40% vs. LVEF≥40%	1.81 (1.07–3.05)	0.027
Hypoalbuminemia vs. no hypoalbuminemia	2.62 (1.65–4.17)	<0.001
Hypertension vs. no hypertension	2.26 (1.41–3.61)	<0.001
Hypotension vs. no hypotension	5.16 (2.46–10.80)	<0.001
Anemia vs. no anemia	2.04 (1.37–3.03)	<0.001
AMI vs. no AMI	4.08 (2.72–6.10)	<0.001
Diabetes vs. no diabetes	1.57 (1.04–2.38)	0.034
ACEI/ARB vs. no ACEI/ARB	0.58 (0.35–0.96)	0.034
Beta blocker vs. no beta blocker	0.27 (0.17–0.41)	<0.001
Diuretic vs. no diuretic	3.54 (2.37–5.29)	<0.001
IABP vs. no IABP	10.00 (5.74–17.40)	<0.001
PCI vs. no PCI	1.48 (0.89–2.48)	0.131
Hydration volume, mL	1.00 (1.00–1.00)	<0.001
Contrast volume, mL	1.00 (1.00–1.00)	0.392
Contrast volume ≥ 100 mL vs. contrast volume < 100 mL	0.94 (0.60–1.48)	0.795
Contrast volume ≥ 200 mL vs. contrast volume < 200 mL	0.99 (0.60–1.63)	0.964

CI-AKI: contrast-induced acute kidney injury; HR: heart rate; DBP: diastolic blood pressure; CKD: chronic kidney disease; SCr: serum creatinine; eGFR: estimate glomerular filtration rate; LVEF: left ventricular ejection fraction; AMI: acute myocardial infarction; ACEI: angiotensin-converting enzymes inhibitors; ARB: angiotensin-receptor blockers; PCI: percutaneous coronary intervention; and IABP: intra-aortic balloon pump.

**Table 3:** Multivariable logistic regression analysis of CI-AKI.

Variable	*β*	OR (95% CI)	*P* value
Age, years	0.04	1.04 (1.02–1.06)	0.001
IABP vs. no IABP	1.38	3.97 (2.14–7.37)	<0.001
AMI vs. no AMI	1.18	3.27 (2.09–5.10)	<0.001
CKD vs. no CKD	1.04	2.83 (1.80–4.44)	<0.001

CI-AKI: contrast-induced acute kidney injury; IABP: intra-aortic balloon pump; AMI: acute myocardial infarction; and CKD: chronic kidney disease.

## Data Availability

Data relevant to this study are available from the corresponding authors upon reasonable request.

## Supplementary Materials

Supplementary Materials
*Supplementary Table 1*. Baseline characteristics. *Supplementary Table 2*. C-statistic and Hosmer–Lemeshow statistic of the nomogram in patients with different stages of congestive heart failure and stages of chronic kidney disease. *Supplementary Figure 1*. The distribution of the serum creatinine value within 24–72 hours after the procedure. *Supplementary Figure 2*. Risk of contrast-induced acute kidney injury according to the total nomogram score categories.
