# Supplementary material for: A Simple Nomogram to Predict Contrast-Induced Acute Kidney Injury in Patients with Congestive Heart Failure Undergoing Coronary Angiography
Source: Cardiol Res Pract. 2021 Mar 23;2021:9614953. doi: 10.1155/2021/9614953 (PMC8009707; doi:10.1155/2021/9614953)

Supplementary table 1. Baseline characteristics

Variables	Total (n = 1876)	Missing value, n (%)	Development cohort (n = 1261)	Validation cohort (n = 615)	p value
Age, years	64.77 ± 10.70	0 (0)	64.71 ± 10.50	64.89 ± 11.11	0.741
Age ≥ 65 years, n (%)	1012 (53.94)	0 (0)	664 (52.66)	348 (56.59)	0.109
Age ≥ 75 years, n (%)	369 (19.67)	0 (0)	244 (19.35)	125 (20.33)	0.618
Female sex, n (%)	471 (25.11)	0 (0)	314 (24.90)	157 (25.53)	0.769
Weight, kg	64.39 ± 10.87	13 (0.69)	64.51 ± 10.85	64.15 ± 10.91	0.505
SBP, mmHg	130.26 ± 21.03	3 (0.16)	130.60 ± 21.17	129.55 ± 20.75	0.305
DBP, mmHg	76.07 ± 12.09	4 (0.21)	76.29 ± 12.06	75.63 ± 12.14	0.015
HR, bpm	75.32 ± 14.17	3 (0.16)	75.30 ± 14.09	75.61 ± 13.95	0.652
Medical history					
Hypotension, n (%)	54 (2.89)	5 (0.27)	36 (2.86)	18 (2.93)	0.935
CKD, n (%)	442 (23.56)	0 (0)	293 (23.24)	149 (24.23)	0.635
CKD stages		0 (0)			0.100
CKD G1, n (%)	555 (29.58)		372 (29.50)	183 (29.76)	
CKD G2, n (%)	879 (46.86)		596 (47.26)	283 (46.02)	
CKD G3, n (%)	385 (20.52)		262 (20.78)	123 (20.00)	
CKD G4, n (%)	49 (2.61)		29 (2.30)	20 (3.25)	
CKD G5, n (%)	8 (0.43)		2 (0.16)	6 (0.98)	
LVEF, %	56.76 ± 13.16	228 (12.15)	56.86 ± 13.25	56.55 ± 12.96	0.655
LVEF < 40%, n (%)	209 (12.68)	228 (12.15)	143 (12.72)	66 (12.60)	0.943

Heart function (NYHA class)		0 (0)			0.444
II, n (%)	1162 (61.94)		770 (61.06)	392 (63.74)	
III, n (%)	158 (8.42)		112 (8.88)	46 (7.48)	
IV, n (%)	29 (1.55)		18 (1.43)	11 (0.87)	
Heart function (Killip class)		0 (0)			0.333
II, n (%)	417 (22.23)		292 (23.16)	125 (20.33)	
III, n (%)	71 (3.78)		44 (3.49)	27 (4.39)	
IV, n (%)	39 (2.08)		25 (1.98)	14 (2.28)	
Hypertension, n (%)	1151 (61.39)	1 (0.05)	788 (62.54)	363 (59.02)	0.142
Hyperlipidemia, n (%)	252 (13.43)	0 (0)	182 (14.43)	70 (11.38)	0.069
Hypoalbuminemia, n (%)	811 (47.34)	163 (8.69)	539 (46.75)	272 (48.57)	0.478
Anemia, n (%)	661 (35.73)	26 (1.39)	432 (34.84)	229 (37.54)	0.254
AMI, n (%)	527 (28.23)	9 (0.48)	361 (28.72)	166 (27.21)	0.498
Diabetes, n (%)	497 (26.51)	1 (0.05)	331 (26.25)	166 (27.04)	0.717
CAD, n (%)	1743 (93.31)	8 (0.43)	1172 (93.39)	571 (93.15)	0.847
		Laboratory examination			
LDL-C, mmol/L	2.69 ± 0.94	318 (16.95)	2.69 ± 0.96	2.70 ± 0.91	0.964
HDL-C, mmol/L	1.01 ± 1.74	318 (16.95)	1.04 ± 2.12	0.95 ± 0.25	0.187
SCr, µmol/L	96.86 ± 50.73	0 (0)	95.51 ± 45.86	99.64 ± 59.43	0.129

eGFR, mL/min/1.73 mm^2	77.40 ± 25.84	0 (0)	77.53 ± 25.23	77.15 ± 27.06	0.774
Hemoglobin, g/L	131.68 ± 16.94	114 (6.08)	132.20 ± 17.13	130.61 ± 16.49	0.06
HbA1c, %	6.60 ± 1.34	416 (22.17)	6.62 ± 1.38	6.57 ± 1.25	0.471
Medications					
ACEI/ARB, n (%)	1636 (87.21)	0 (0)	1091 (86.52)	545 (88.62)	0.201
Beta blocker, n (%)	1570 (83.73)	1 (0.05)	1060 (84.13)	510 (82.93)	0.509
Statin, n (%)	1797 (95.84)	1 (0.05)	1215 (96.35)	582 (94.79)	0.112
Diuretics, n (%)	428 (22.83)	1 (0.05)	288 (22.86)	140 (22.76)	0.964
Procedure					
PCI, n (%)	1234 (69.44)	99 (5.28)	853 (71.08)	381 (66.03)	0.030
Hydration volume, mL	813.27 ± 472.63	32 (1.71)	816.77 ± 467.72	806.08 ± 482.88	0.652
Contrast volume, mL	131.82 ± 67.17	0 (0)	134.38 ± 68.22	126.58 ± 64.70	0.016
Contrast volume≥ 100mL, n (%)	1393 (74.25)	0 (0)	950 (75.34)	443 (72.03)	0.124
Contrast volume≥ 200mL, n (%)	343 (18.28)	0 (0)	245 (19.43)	98 (15.93)	0.066
Mehran Score	5.24 ± 4.66	30 (1.60)	5.15 ± 4.60	5.44 ± 4.78	0.220
Peri-procedure IABP, n (%)	99 (5.28)	0 (0)	61 (4.84)	38 (6.18)	0.223

Abbreviations: CI-AKI: contrast-induced acute kidney injury; SBP: systolic blood pressure; DBP: diastolic blood pressure; HR: heart rate; LVEF: left ventricular ejection fraction; CKD: chronic kidney disease; NYHA: New York Heart Association; AMI : acute myocardial infarction; CAD: coronary artery disease; LDL-C: low density lipoprotein-C; HDL-C: high density lipoprotein-C; SCr: serum creatinine; eGFR: estimate glomerular filtration rate; ACEI: angiotensin-converting enzymes inhibitors; ARB: angiotensin-receptor blockers; PCI: percutaneous coronary intervention; IABP: intra-aortic balloon pump;

Supplementary table 2. C-Statistic and Hosmer-Lemeshow statistic of the nomogram in patients with different stages of congestive heart failure and stages of chronic kidney disease

Stages	C-statistic (95% CI)	Hosmer-Lemeshow statistic	
		$\chi 2$	P value
CKD G1	0.736 (0.638 - 0.834)	7.362	0.498
CKD G2	0.777 (0.713 – 0.840)	6.961	0.541
CKD G3	0.708 (0.636 – 0.779)	6.005	0.647
CKD G4&5	0.699 (0.559 – 0.839)	4.344	0.739
Heart function (NYHA/Killip class)			
II	0.797 (0.754 – 0.840)	3.525	0.897
III	0.745 (0.650 – 0.839)	12.752	0.121
IV	0.647 (0.510 – 0.783)	12.966	0.113

Abbreviations: CKD: chronic kidney disease; NYHA: New York Heart Association;

Supplementary Figure 1. The distribution of the serum creatinine value within 24-72 hours after the procedure

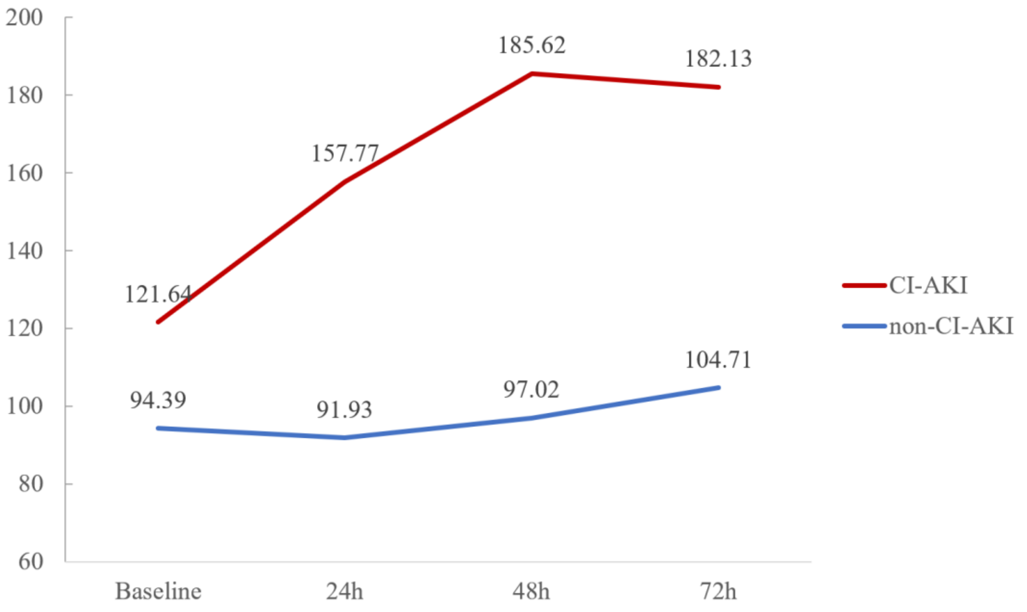

	**24h**	**48h**	**72h**
SCr missing in CI-AKI group	20.59 %	17.06 %	64.12 %
SCr missing in non-CI-AKI group	9.20 %	51.82 %	86.69 %

Supplementary Figure 2. Risk of contrast-induced acute kidney injury according to the total nomogram score categories.

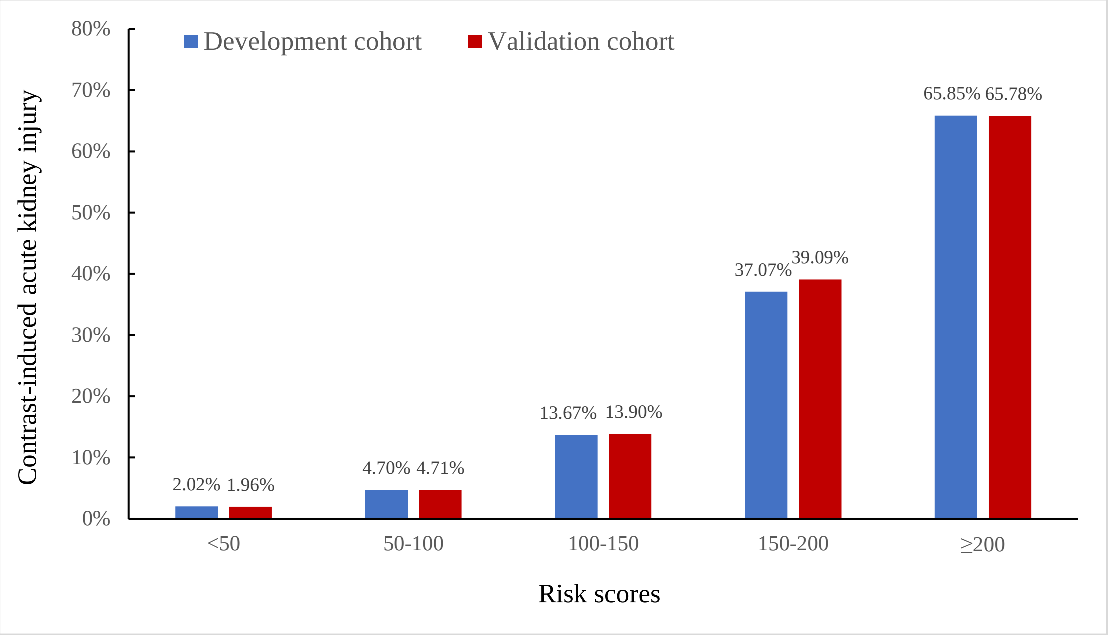

Supplement: Supplementary Materials — Supplementary Table 1. Baseline characteristics. Supplementary Table 2. C-statistic and Hosmer–Lemeshow statistic of the nomogram in patients with different stages of congestive heart failure and stages of chronic kidney disease. Supplementary Figure 1. The distribution of the serum creatinine value within 24–72 hours after the procedure. Supplementary Figure 2. Risk of contrast-induced acute kidney injury according to the total nomogram score categories. [file 9614953.f1.pdf]
